# Awake Plastic Surgery Procedures: The Use of a Sufentanil Sublingual Tablet to Improve Patient Experience

**DOI:** 10.1093/asjof/ojab056

**Published:** 2022-01-27

**Authors:** Hisham Seify

## Abstract

**Background:**

Awake plastic surgery performed under minimal sedation has advantages including patient preference, affordability, and easier recovery compared to when performed under deeper sedation. Commonly used oral analgesics may not be adequate for awake procedures resulting in moderate to severe pain. Sufentanil sublingual tablet (SST) 30 mcg has been shown to provide timely analgesia with a safety profile appropriate for minimal-sedation settings.

**Objectives:**

To examine perioperative outcomes in patients who underwent awake plastic surgery with local anesthesia and SST 30 mcg for pain control.

**Methods:**

This study was a prospective single-group cohort study conducted at a single plastic surgery center. SST 30 mcg was administered approximately 30 minutes prior to the procedure. Outcome measures included the number of patients with adverse events, the number of patients requiring medications in the post-anesthesia care unit (PACU), and recovery time.

**Results:**

Among the 31 patients, the most common procedures were liposuction (71%), facelift (10%), and blepharoplasty (6%). The mean (± standard error [SE]) procedural duration was 81 ± 9 minutes. No vital sign instability or oxygen desaturation was observed. Three patients (10%) experienced nausea, only one of which required treatment with oral ondansetron 4 mg in the PACU. One patient (3%) experienced dizziness that did not require treatment. No patients required opioids or other analgesics in the PACU for pain. The mean (±SE) recovery time was 15 ± 4 minutes.

**Conclusions:**

Awake plastic surgery can be performed using SST 30 mcg with minimal side effects and a rapid recovery time.

**Level of Evidence: 4:**

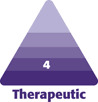

Plastic surgery procedures including liposuction and facial procedures can be performed with deep sedation or general anesthesia^1,2^; however, there has been an increasing trend for these procedures to be performed with local anesthesia and minimal sedation, in part due to patient preference for awake procedures.^3-5^ Minimal sedation is the lowest level of sedation along the continuum of sedation/analgesia described by the American Society of Anesthesiologists (Table 1).^6^ With minimal sedation, patients respond normally to verbal commands. Cognitive function and physical coordination may be affected. However, airway reflexes, spontaneous ventilation, and cardiovascular function are unaffected. Minimal sedation can offer several advantages over deeper levels of sedation for various plastic surgery procedures. It reduces the cost of procedures compared with deep sedation or general anesthesia because of lower resource utilization.^7^ Minimal sedation also helps to reduce the risk of serious adverse events associated with general anesthesia and deep sedation, which can include cardiovascular and respiratory complications.^8-11^ Furthermore, minimal sedation promotes an easier recovery than deeper levels of sedation.^7^ This is in part because nausea and vomiting are more common with deeper sedation and general anesthesia,^7,12^ contributing to longer recovery times, unintended hospital admissions, and complications such as hematoma and incisional dehiscence.^7,12-14^ Patients may prefer minimal sedation because of affordability, convenience, and fears of general anesthesia or deep sedation.^3,7,15^ Older patients may prefer minimal sedation because of the desire to avoid postoperative delirium and the possible association between anesthesia and cognitive decline.^9,16^

**Table 1.** Levels of Sedation/Analgesia

	Minimal sedation/anxiolysis	Moderate sedation/analgesia (“conscious sedation”)	Deep sedation/analgesia	General anesthesia
*Responsiveness*	Normal response to verbal stimulation	Purposeful^a^ response to verbal or tactile stimulation	Purposeful^a^ response following repeated or painful stimulation	Unarousable even with painful stimulus
*Airway*	Unaffected	No intervention required	Intervention may be required	Intervention often required
*Spontaneous Ventilation*	Unaffected	Adequate	May be inadequate	Frequently inadequate
*Cardiovascular Function*	Unaffected	Usually maintained	Usually maintained	May be impaired

All procedures in the present study were planned for the minimal-sedation setting. Excerpted from Continuum of Depth of Sedation: Definition of General Anesthesia and Levels of Sedation/Analgesia 2019 of the American Society of Anesthesiologists. A copy of the full text can be obtained from ASA, 1061 American Lane, Schaumburg, IL 60173-4973 or online at www.asahq.org.

^a^Reflex withdrawal from a painful stimulus is NOT considered a purposeful response.

Given its advantages, there is interest in using minimal sedation with a variety of plastic surgery procedures, including those that may result in substantial pain. Local anesthetic infiltration may provide sufficient analgesia for certain limited awake procedures. However, more extensive and invasive procedures often require additional pain control. Adequate pain management is important so that the awake surgery is not disrupted, and the patient has a positive perioperative experience. Patients who are comfortable during the procedure may be more willing to return to the clinic for additional procedures and may recommend the clinic to other potential patients. Adequate acute pain management is also important for avoiding the development of chronic pain.^17,18^

Oral opioids, which may be used as a pretreatment for awake plastic surgery, are limited in that they have a relatively slow onset.^19-21^ This could increase the duration of time required for the patient to be at the clinic or require the patient to self-medicate before arriving at the clinic, both of which may be inconvenient, and the effectiveness of the latter depends on the reliability of the patient. An easily administered opioid with a rapid onset of action that has a side-effect profile consistent with the criteria for minimal sedation could be advantageous for awake plastic surgery procedures.

Sufentanil sublingual tablet (SST) 30 mcg (DSUVIA; AcelRx Pharmaceuticals, Hayward, CA) is a novel opioid analgesic that was approved by the US Food and Drug Administration (FDA) and the European Medicines Agency in 2018 for the management of acute pain severe enough to require an opioid in medically supervised healthcare settings.^22-24^ It is currently the only FDA-approved sublingual drug for acute pain management. SST 30 mcg has a rapid analgesic onset of 15 to 30 minutes, a peak plasma concentration at 1 hour, and a duration of approximately 3 hours.^25-28^ In addition, SST 30 mcg has been shown to have minimal effects on cognitive and respiratory function.^29,30^ Cognitive impairment was assessed in a study using the 6-Item Screener^31^ before and 1 hour after dosing with SST 30 mcg.^29^ In that study, 97% of patients had either the same or an improved cognitive score at 1 hour,^29^ which is the time of expected maximal effect of SST 30 mcg.^28^ Furthermore, in phase 3 studies, no patient required naloxone with SST 30 mcg administered for up to 48 hours.^26,27,29,30^ These safety findings suggest that SST 30 mcg is appropriate for pain management in settings utilizing minimal-sedation protocols, especially when administered as a single dose. In this study, we evaluated SST 30 mcg for pain management for awake plastic surgery procedures planned for the minimal-sedation setting, and we examined perioperative outcomes including adverse events and recovery time.

## METHODS

### Study Design

This study was a prospective single-group cohort study conducted from January 2020 through October 2021 at an ambulatory surgery center certified by the American Association for Accreditation of Ambulatory Surgery Facilities (AAAASF). The study protocol was approved by an independent institutional review board (WIRB-Copernicus Group; WCG IRB #1-402466-1), and all patients provided written informed consent before participation. The study adhered to the ethical principles of the Declaration of Helsinki.

### Eligibility Criteria

Males and females ≥18 years of age scheduled to undergo awake plastic surgery procedures that were expected to result in moderate to severe perioperative pain (eg, liposuction) were eligible for inclusion. Exclusion criteria were known severe reaction to opioids or severe chronic pulmonary disease. Patients were required to have a ride home after the procedure.

### Protocol for Surgery With Minimal Sedation Using SST 30 mcg

**Figure 1.**
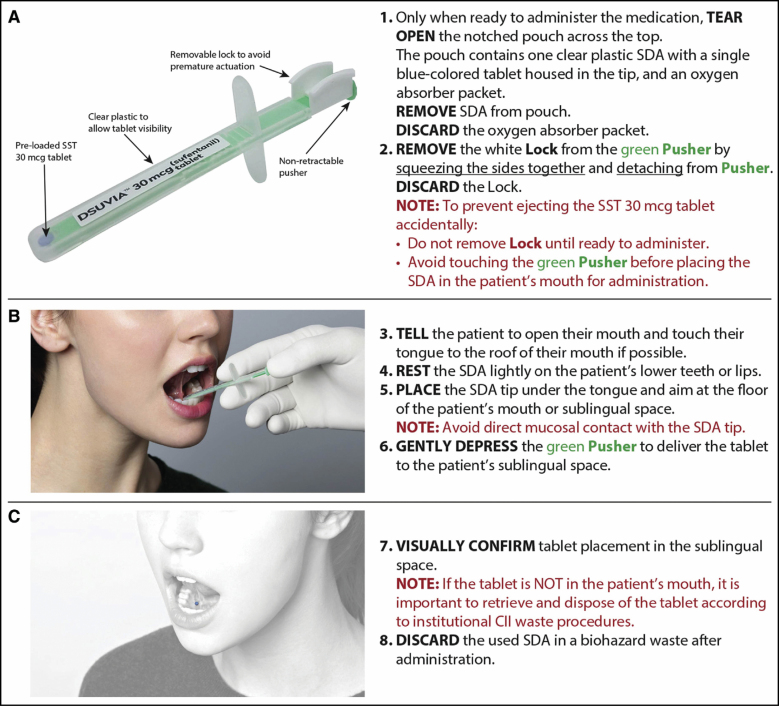
Instructions for the administration of SST 30 mcg. (A) Prepare the SDA for administration of SST 30 mcg. (B) Administer SST 30 mcg in the patient’s mouth with the SDA. (C) Confirm SST 30 mcg placement in the patient’s mouth. CII, class II; SDA, single-dose applicator; SST, sufentanil sublingual tablet. Reprinted from *J Perianesth Nurs*, 35(1), Hutchins JL, Leiman D, Rafique Z, DiDonato KP, Palmer PP, Pooled dosing and efficacy analysis of the sufentanil sublingual tablet 30 mcg across demographic subgroups for the management of moderate-to-severe acute pain, 22-28; Copyright (2019), with permission from Elsevier.

Ondansetron 4 mg PO (by mouth) was administered with sips of water as antiemetic prophylaxis just before SST 30 mcg administration. SST 30 mcg was then administered sublingually by the healthcare provider approximately 30 minutes before the procedure using the prefilled single-dose applicator (Figure 1). The patient was instructed to minimize talking for at least 5 minutes after SST 30 mcg administration to allow full absorption of the tablet. Benzodiazepines were to be given at half of the regular dose to patients already taking benzodiazepines on a chronic basis. The choice of infiltration of local anesthesia depended on the procedure. When tumescence was used, the tumescent fluid consisted of 1000 mL of lactated ringer’s solution, 1 mL of epinephrine (1:1000), and 10 mL of sodium bicarbonate, with or without 1000 mg of tranexamic acid. Approximately, 80 to 100 mL of lidocaine 1% was added per liter. The amount of infiltrated tumescent fluid ranged from approximately 500 to 3500 mL. For less extensive cases, local infiltration with either lidocaine 1% or bupivacaine 0.5% with epinephrine was used.

Supplemental oxygen, a bag valve mask (eg, Ambu bag), and naloxone were available, if necessary, to treat respiratory depression.^22^ Vital signs (pulse rate, respiratory rate, blood pressure, and oxygen saturation) were recorded before and after the procedure. Pulse rate and oxygen saturation were monitored continuously throughout the procedure and recovery, and respiratory rate and blood pressure were measured periodically.

The possibility to redose with SST 30 mcg for inadequate analgesia was available, with a minimum of 1 hour between doses.^22^ Following the procedure, the patient was instructed to use a nonprescription nonsteroidal anti-inflammatory drug (NSAID) or acetaminophen for the treatment of postoperative pain.

### Outcome Measures

The procedure beginning and end times, adverse events, medications received during the procedure and in the postanesthesia care unit (PACU), and time of discharge were recorded. Outcome measures included the number of patients with adverse events (including any vital sign instability), the number of patients requiring medications during the procedure or in the PACU, and the recovery time, defined as the duration from the procedure end time to discharge. All mean values are reported as mean ± standard error (SE).

## RESULTS

### Patients and Procedures

A total of 31 patients (28 females and 3 males) were enrolled and underwent awake plastic surgery procedures with a preoperative dose of SST 30 mcg and local anesthesia infiltration (Table 2). No patients enrolled were taking chronic outpatient benzodiazepines. Therefore, these medications were not administered per protocol. The average age of the patients was 47.4 ± 2.2 years, with a range of 23 to 71 years. All patients received one dose of SST 30 mcg, with no patients requiring a second dose for inadequate analgesia. The majority of procedures conducted were liposuction (71%; 22/31), facelift (10%; 3/31), and blepharoplasty (6%; 2/31). The remaining patients underwent fat transfer, radiofrequency microneedling, abdominal incision and drainage, and nasal reconstruction. The average procedure duration was 81 ± 9 minutes (range, 20-198 minutes).

**Table 2.** Summary of Patient Demographics, Procedures, Adverse Events, and Medications Received in the PACU

	Patients who received SST 30 mcg (N = 31)
Patient demographics	
Age, years, mean (SE)	47.4 (2.2)
Female, n (%)	28 (90%)
Procedure	
Liposuction, n (%)	22 (71%)
Facelift, n (%)	3 (10%)
Blepharoplasty, n (%)	2 (6%)
Other, n (%)	4 (13%)
Procedure duration, minutes, mean (SE)	81 (9)
Recovery time, minutes, mean (SE)	15 (4)
Adverse events	
Nausea, n (%)	3 (10%)
Dizziness, n (%)	1 (3%)
Medications received in the PACU	
Antiemetic, n (%)	1 (3%)

PACU, postanesthesia care unit; SE, standard error; SST, sufentanil sublingual tablet.

### Efficacy

All 31 patients completed the plastic surgery procedures successfully without disruption from inadequate analgesia. Aside from the single preoperative dose of SST 30 mcg and local anesthetic infiltration, no other opioids or other analgesics were provided intraoperatively or in the PACU. The average recovery time was 15 ± 4 minutes (range, 0-85 minutes).

### Safety

Oxygen saturation did not decrease below 95% during the procedure or recovery period for any of the 31 patients, and no supplemental oxygen was required. Vital signs were stable during the procedures and in recovery. Three patients (10%) experienced nausea, only one of whom required treatment with oral ondansetron 4 mg in the PACU. One patient (3%) experienced dizziness that did not require treatment. No patient experienced vomiting.

## Discussion

This study evaluated perioperative outcomes in patients who received SST 30 mcg for pain control for plastic surgery procedures planned for the minimal-sedation setting. For a drug-induced state to be considered minimal sedation, patients in that state must respond normally to verbal commands, and airway reflexes, spontaneous ventilation, and cardiovascular function must be unaffected.^6^ This contrasts with higher levels of sedation including moderate sedation (ie, conscious sedation), in which there is a depression of consciousness and the patient responds “purposefully” to verbal or tactile stimulation, spontaneous ventilation is adequate, cardiovascular function is usually maintained, and no intervention is required for airway (Table 1). In the present study, no respiratory or cardiovascular events were observed with SST 30 mcg, and no supplemental oxygen was required. Furthermore, a previous study suggests that SST 30 mcg has little to no effect on cognitive function.^29^ These findings suggest that SST 30 mcg is appropriate for providing pain management under minimal-sedation guidelines. The present study also found that patients had a rapid recovery time, with no additional analgesics administered in the PACU. This case series included fairly extensive awake surgical procedures as well as more minor aesthetic procedures including radiofrequency microneedling, which can cause significant pain even though it is relatively noninvasive. Regardless of the procedure, patients benefited from the analgesia provided by SST 30 mcg, with a low rate of side effects.

Although SST 30 mcg has been found to be generally well tolerated, safety precautions are needed when using SST 30 mcg, as opioids can result in life-threatening respiratory depression.^32^ SST 30 mcg must be administered by a healthcare provider in a supervised healthcare setting.^22^ Healthcare settings need to be certified by a Risk Evaluation and Mitigation Strategy (REMS) program as required by the FDA.^33^ Patients administered SST 30 mcg should be closely monitored for respiratory depression, and healthcare providers must be ready to manage complications of overdose.^22^

**Figure 2.**
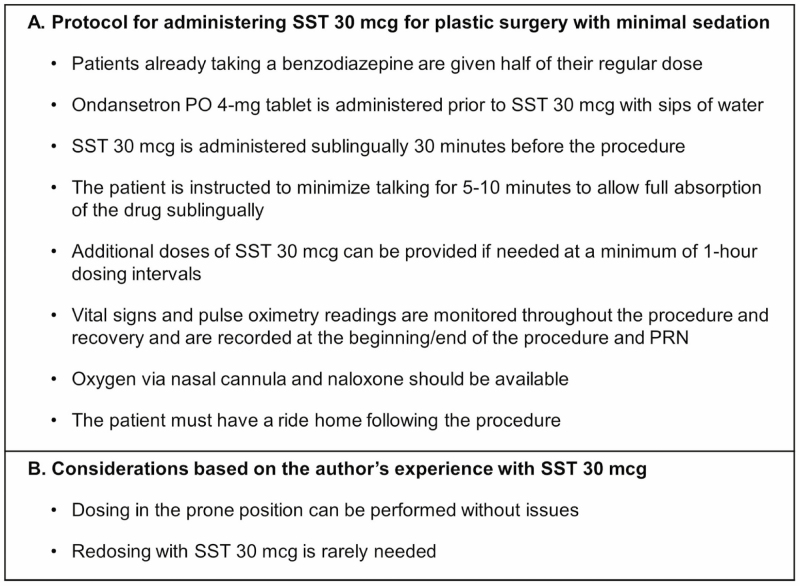
Recommended protocol and considerations. (A) Recommended protocol for administering SST 30 mcg for pain management for plastic surgery procedures with minimal sedation. (B) The experience of the author with SST 30 mcg for pain management for plastic surgery procedures with minimal sedation, outside of the present study. PO, by mouth; PRN, as needed; SST, sufentanil sublingual tablet.

The protocol presented in Figure 2A is recommended by the author for using SST 30 mcg for pain management for plastic surgery performed in the minimal-sedation setting. Based on the experience of the author, redosing of SST 30 mcg is rarely needed, and patient comfort is improved compared with procedures performed with local anesthesia alone or with an oral opioid (Video). In this patient series, our observations demonstrated that SST 30 mcg allowed the patient to be not only comfortable but also conversant, even during procedures over an hour in length. All procedures were able to be completed without additional analgesics. The average recovery time of 15 minutes from the PACU likely reflects the lack of cognitive impairment demonstrated in a preapproval clinical trial, the low incidence of side effects, and the effective pain management.^29^

**Figure 3.**
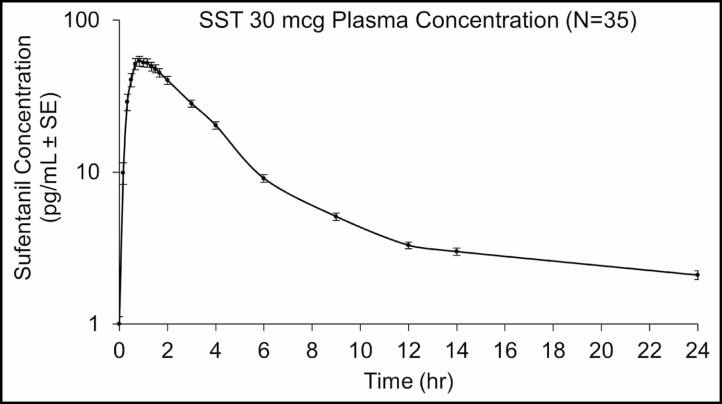
Plasma concentrations of sufentanil after SST 30 mcg administration. SE, standard error; SST, sufentanil sublingual tablet. Reproduced with permission from Fisher DM, Chang P, Wada DR, Dahan A, Palmer PP. Pharmacokinetic properties of a sufentanil sublingual tablet intended to treat acute pain. *Anesthesiology*. 2018;128(5):943-952. https://doi.org/10.1097/ALN.0000000000002145.

Despite the requirement for supplemental oxygen and naloxone to be readily available during the procedure, the author has not had to use either of these medications on any patient who has received SST 30 mcg. In addition, dosing of SST in patients in the prone position using the single-dose applicator has been performed successfully (Figure 2B). In the present study, a nonprescription NSAID or acetaminophen was recommended as needed to patients for postoperative pain. Because the terminal half-life of SST 30 mcg is 13 hours,^28^ a prolonged analgesic tail may contribute to pain relief in combination with these drugs (Figure 3).

Other agents not used in the present study that have been reported to increase patient comfort during awake procedures include an inhaled mixture of nitrous oxide and oxygen as well as a sublingual tablet combining midazolam 3 mg, ketamine hydrochloride (HCl) 25 mg, and ondansetron 2 mg available from compounding pharmacies but not FDA-approved.^34-36^ Mixtures of nitrous oxide and oxygen can be used for mild analgesia and anxiolysis without loss of consciousness.^34,35^ Advantages of nitrous oxide include that it has a rapid onset and offset and a long history of demonstrated safety.^34,37^ Limitations include that it is a relatively mild analgesic and is, therefore, often combined with other systemic analgesics or a nerve block.^34,37,38^ In addition, administration of nitrous oxide involves using a mask or mouthpiece,^34^ which may interfere with facial procedures. The sublingual tablet combination of midazolam 3 mg as a sedative, ketamine HCl 25 mg as an analgesic and sedative, and ondansetron 2 mg as an antiemetic has been studied for use during cataract surgery.^36,39^ While the midazolam dose should be sufficient to produce sedation,^40,41^ the doses of ketamine and ondansetron are likely in the subtherapeutic range as both of these agents undergo metabolism when administered through the sublingual route, resulting in an approximate 30% and 56% bioavailability, respectively.^42,43^ The resultant doses of 7.5 mg ketamine and 1.1 mg ondansetron are below the lower end of efficacy for these medications.^43,44^

There were several limitations with the present study. A small sample size was evaluated, and there was no control group of patients who did not receive SST 30 mcg. Because of the lack of a control group, the effect of SST 30 mcg on perioperative pain scores was not directly evaluated. Data collection is ongoing to examine outcomes in a larger number of patients.

## Conclusions

Awake surgical procedures performed under minimal sedation can offer advantages over those performed with higher levels of sedation, including patient preference, affordability, reduced medical risks, and easier recovery.^3,7,8^ SST 30 mcg is a novel analgesic option to provide timely pain control to patients undergoing awake plastic surgery. In this study of 31 patients undergoing plastic surgery procedures with local anesthesia and SST 30 mcg, minimal adverse events occurred, and the average recovery time was 15 minutes. These findings support that SST 30 mcg is appropriate for the minimal-sedation setting.
